# Report of the Post Kala-Azar Dermal Leishmaniasis (PKDL) consortium meeting, New Delhi, India, 27–29 June 2012

**DOI:** 10.1186/1756-3305-6-196

**Published:** 2013-07-02

**Authors:** Philippe Desjeux, Raj Shankar Ghosh, Pritu Dhalaria, Nathalie Strub-Wourgaft, Ed E Zijlstra

**Affiliations:** 1 PATH OWH, A-9, Qutub Institutional area, USO Road, New Delhi 110067, India; 2 DNDi, 15 Chemin Louis Dunant, Geneva 1202, Switzerland

**Keywords:** Consortium report, PKDL, Post kala-azar dermal leishmaniasis, Visceral leishmaniasis

## Abstract

Post kala-azar dermal leishmaniasis (PKDL) is a neglected complication of visceral leishmaniasis (VL)―a deadly, infectious disease that claims approximately 20,000 to 40,000 lives every year. PKDL is thought to be a reservoir for transmission of VL, thus, adequate control of PKDL plays a key role in the ongoing effort to eliminate VL. Over the past few years, several expert meetings have recommended that a greater focus on PKDL was needed, especially in South Asia. This report summarizes the Post Kala-Azar Dermal Leishmaniasis Consortium Meeting held in New Delhi, India, 27–29 June 2012. The PKDL Consortium is committed to promote and facilitate activities that lead to better understanding of all aspects of PKDL that are needed for improved clinical management and to achieve control of PKDL and VL. Fifty clinicians, scientists, policy makers, and advocates came together to discuss issues relating to PKDL epidemiology, diagnosis, pathogenesis, clinical presentation, treatment, and control. Colleagues who were unable to attend participated during drafting of the consortium meeting report.

## Introduction

**Figure 1:**
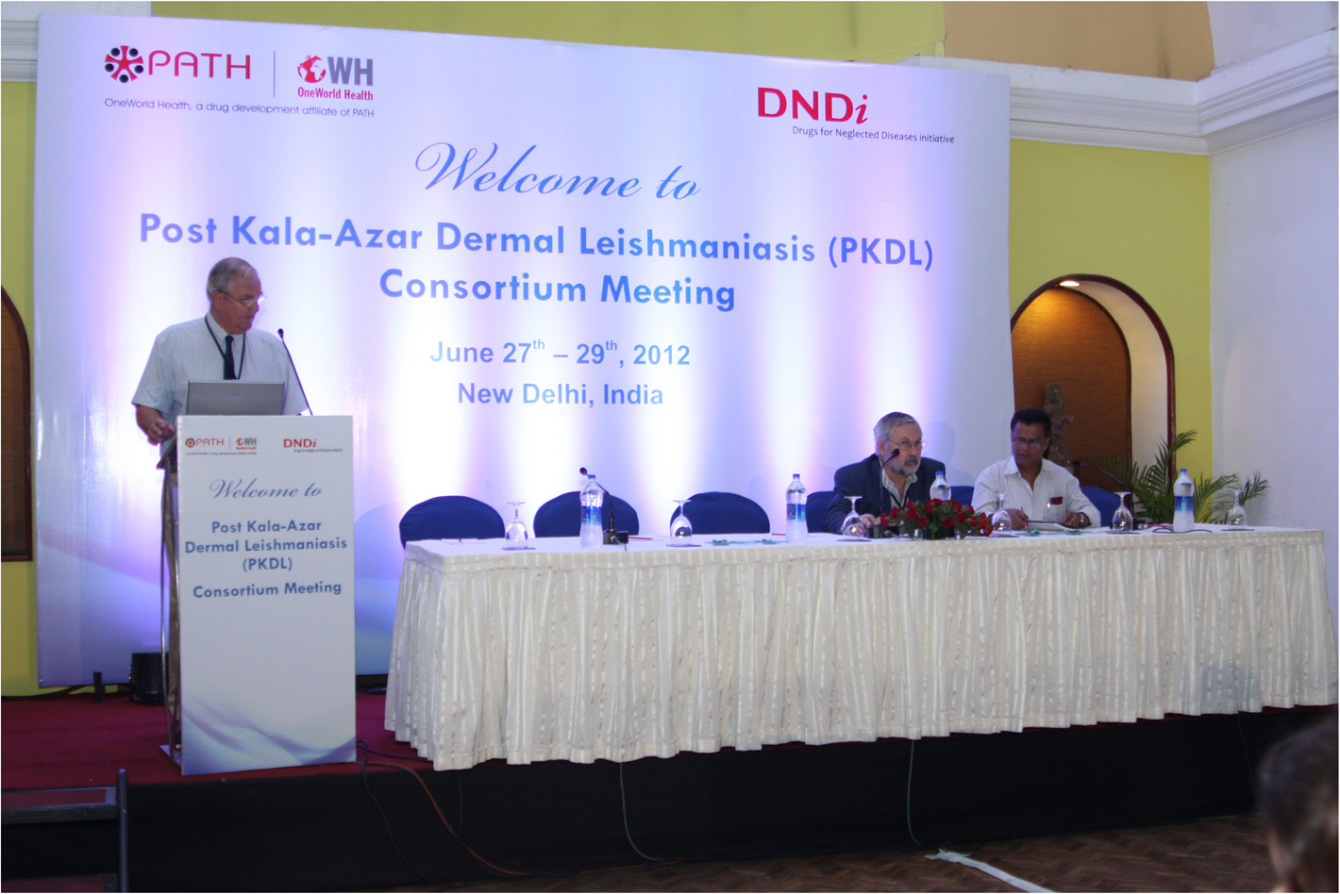
The PKDL Consortium meeting in New Delhi, 27-29 June 2013.

**Figure 2:**
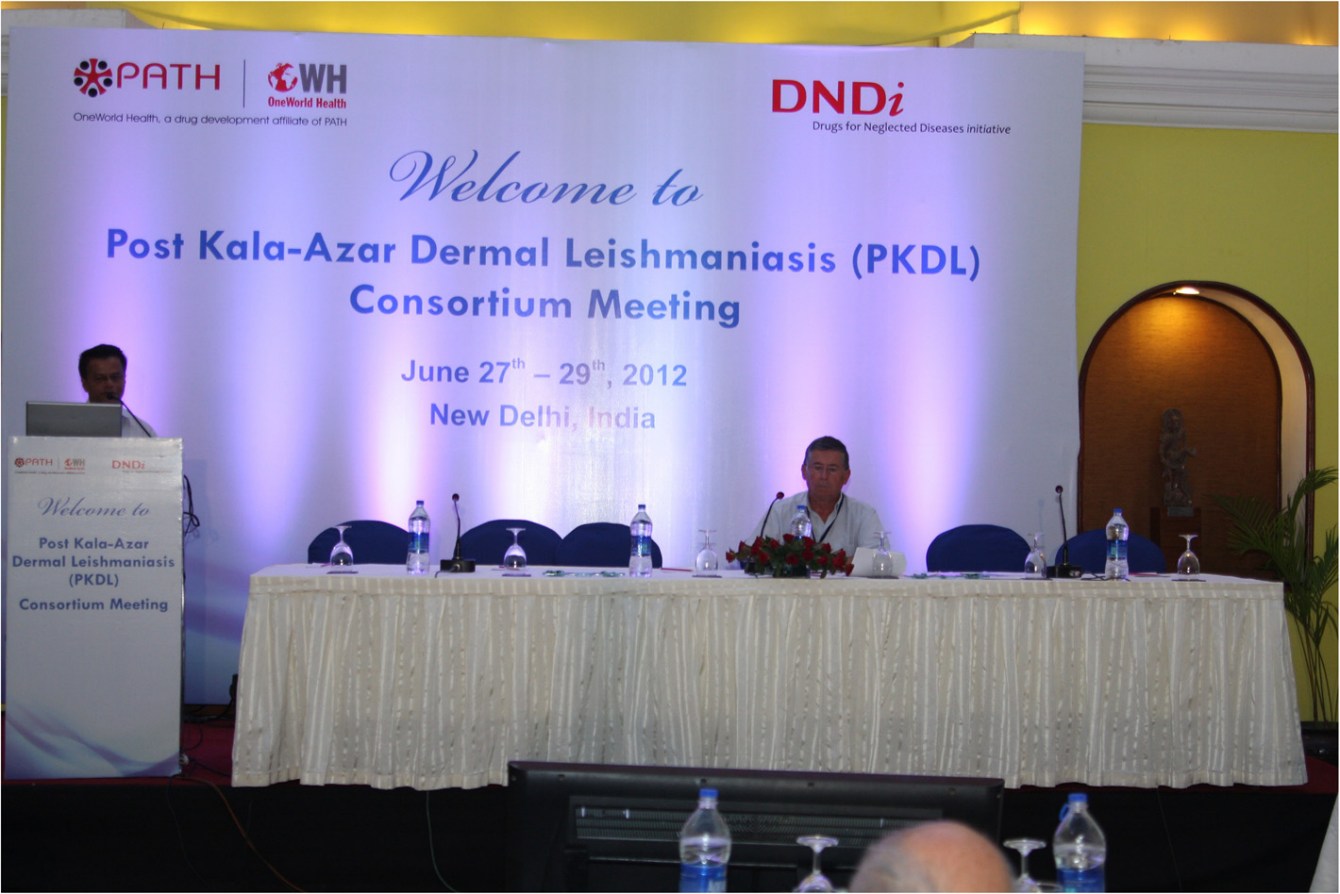
The PKDL Consortium meeting in New Delhi, 27-29 June 2013.

Post kala-azar dermal leishmaniasis (PKDL) is a neglected complication of visceral leishmaniasis (VL, also known as kala-azar or black fever)―a deadly, infectious disease that claims approximately 20,000 to 40,000 lives every year [1]. PKDL is thought to be a reservoir for transmission of VL and is very difficult to treat, especially in some patients in East Africa who suffer from a severe form of PKDL. The PKDL Consortium, which convened in New Delhi, India, on 27–29 June 2012, brought together experts on VL and PKDL to review issues relating to PKDL epidemiology, diagnosis, pathogenesis, clinical presentation, treatment, and control in East Africa and South Asia (Figures 1 and 2).

The opening session was chaired by Dr. Philippe Desjeux and Prof. Ed Zijlstra, with welcoming comments from Dr. V. M. Katoch, Director, Indian Council of Medical Research (ICMR); Dr. Tarun Vij, Country Program Leader, PATH – OneWorld Health (OWH); Dr. Nathalie Strub-Wourgaft, Drugs for Neglected Diseases *initiative* (DND*i*); Dr. J. Alvar, Neglected Tropical Diseases, World Health Organization (WHO), Geneva; and Dr. Lalit Kant, Bill & Melinda Gates Foundation (BMGF). Dr. Philippe Desjeux and Prof. Ed Zijlstra then presented the rationale for the PKDL consortium, which was based on a number of recommendations from the WHO Leishmaniasis Expert Committee and a recent Regional Technical Advisory Group (RTAG) meeting where a lack of tools and research conducted on PKDL was recognized. Following this, OWH and the Leishmaniasis East Africa Platform (LEAP) also recognized the importance of giving PKDL high priority. In January 2012, a concept note was agreed on, by OWH and DND*i* for the development of a PKDL consortium. Following re-purposing of some initial funding by PATH – OWH this consortium was supported together with DND*i*.

The main objective was to develop a research agenda to facilitate improved control of PKDL by bringing together a small technical platform, including partners from key endemic regions for PKDL, e.g., the Institute of Endemic Diseases (IED), Sudan and the International Centre for Diarrhoeal Disease Research, Bangladesh (ICDDR-B), Dhaka, Bangladesh, and to raise substantial funding.

The proposed Mission Statement was as follows: “The PKDL consortium is committed to promoting and facilitating activities that lead to better understanding of all aspects of PKDL that are needed for improved clinical management and to achieve control of PKDL and VL.” The role of the consortium was to establish a PKDL research agenda, fundraising, training, advocacy, communication, and the development of links between East Africa and South Asia to further increase understanding of both VL and PKDL to allow the implementation of new preventive and control measures based on research end-products.

### Presentations

Please see Additional file 1 for the meeting program and Additional file 2 for a list of names of contributors.

### Session 1 Epidemiology

Presentation and discussion sessions on the epidemiology of PKDL were chaired by Prof. N. K. Ganguly, Prof. Ridwanur Rahman and Dr. Monique Wasunna.

#### Asia

Prof. C. P. Thakur described epidemiology in India. There are 4 reasons why VL remains present in India: (1) VL patients, (2) PKDL patients, (3) sand flies, and (4) lack of sustained control activities by the government. The history of PKDL in India was reviewed, and the role of Dr. Brachmachari as the pioneer of PKDL awareness and research was described.

When DDT was used as part of the malaria eradication program, very few cases of VL were reported. At this time, the number of sandflies also declined, as did the number of cases of VL. However, there is no data to indicate the number of PKDL cases. When use of DDT was stopped a few years later, there was an increase in the number of VL cases, which resulted in a large VL epidemic [2]. Progressive resistance of VL to treatment with sodium stibogluconate (SSG) (30% of cases) also caused the number of PKDL cases to increase. While PKDL cases were reported in a limited number of patients treated with miltefosine for VL, only a few cases were reported following VL treatment with amphotericin B. This was illustrated in the 1980s when SSG was massively used and there was an increase in the incidence of PKDL followed by a large VL epidemic. There was an increase in the use of amphotericin B to treat VL, but there was no corresponding increase in the number of cases of PKDL. This is thought to be the result of more efficient VL treatment with amphotericin B [3,4]. In 2011 in India, there was a slight increase in the number of cases of PKDL reported compared with the number of cases seen in previous years. In the 1960s, Dr. Sen Gupta described the slow response to treatment and proposed that PKDL acts as a reservoir and is the reason that VL returned in India [5]. Current treatment for PKDL used by Dr. Thakur is amphotericin B (desoxycholate): 4 courses (1 mg/kg/day for 15 days) separated by 20 days.

Prof. Mohamed Abul Faiz described epidemiology in Bangladesh. VL was first reported from the Indian subcontinent from Jessore in 1824. In the next century, progressive spread followed throughout the region including Bangladesh, and VL was almost eradicated in 1960 due to DDT spraying for malaria. However, a resurgence occurred in the 1990s, and VL is now endemic in 32/64 districts, mostly in central and north Bangladesh, with an estimated total number of 50,000 cases. There is equal distribution between sexes, although a facility-based study showed that 60% to 80% of cases were in males. The highest endemicity is by far in Mymensingh, and within Mymensingh, in Fulbaria and Trishal. In 2011, 3,376 cases and 2 deaths were officially reported in Bangladesh; underreporting is common. Literature on PKDL in Bangladesh is scarce. PKDL was first described in 1922 by Brahmachari [6]. No data on incidence are available; it is assumed that 10% to 15% of VL cases develop PKDL, but true figures may be 4–5 times higher. In a population study, PKDL incidence increased sharply beginning in 2002. In June 2007, in one village in Fulbaria, point prevalence of PKDL was 3.8/1000 population, and the PKDL rate among patients recently treated (with SSG) was 13.9%.

The Surya Kanta Kala-azar Research Center (SKKRC) in Mymensingh reported 29 PKDL patients, of which 24 were skin slit positive. Eighteen of these patients did not complete SSG treatment, and one defaulted. Twenty-four had a history of VL, for which 20 were treated with SSG, three with miltefosine, and one with AmBisome. ICDDR, B prospectively followed 200 patients treated for VL with SSG (20 mg/kg for 21 days), 36 of whom developed PKDL within 2 years. Recently, it was found that the retina is also affected in a proportion of VL patients. There is a knowledge gap in Bangladesh regarding the true rate of PKDL. Surveillance is needed to better ascertain PKDL cases, and improvements in diagnosis and treatment are needed to better control VL.

Prof. Suman Rijal described epidemiology in Nepal. The numbers of VL and PKDL patients in Nepal are much lower than the number of cases seen in India or Bangladesh. The majority of cases are reported in 12 endemic districts where 25% of the population of the country lives [7,8]. The first case of VL was recorded in the 1990s, and the first case of PKDL was recorded in 1998. There was an increase in the number of cases in the late 1990s, but the case load has decreased since 2006. With the exception of a few districts, Nepal is close to meeting the target for VL elimination of less than 1 case per 10,000 population. The Nepalese Government has been providing free drugs to treat VL and PKDL, so very few cases are treated in the private sector. Of note is that PKDL patients may not seek treatment, and when they do, it is more likely to be at tertiary care hospital centers and, very often, at a private sector dermatologist. Data on the number of PKDL cases are limited, and the government has recorded no data. A survey was conducted to investigate the burden of PKDL in 2010 by reviewing records of 742 VL patients, of which 680 were screened for PKDL between 2000 and 2009. Field workers were trained to recognize potential clinical cases and visited the individual houses to ask about skin lesions and VL treatment history. Of the 680 screened patients, 37 suspect PKDL cases were identified, of which 33 attended a hospital for formal diagnosis. Fourteen were diagnosed with confirmed PKDL and 2 were probable cases of PKDL. Patients were mainly adults with a mean age of 23.5 years. The risk of developing PKDL following treatment with SSG was 1.2%, 2.5%, and 3.6% for an interval after VL of 0 to <2, 2 to <4, and 4 to <8 years, respectively. This low prevalence could be due to good compliance to treatment, as there was a higher risk of developing PKDL if the patients had not completed VL treatment [9]. The prevalence of PKDL may be higher in districts that are close to districts with demonstrated high resistance to SSG. Passive surveillance will not identify PKDL cases; active case finding will be imperative to meet the goal of the elimination program.

#### Africa

Prof. Ed Zijlstra described epidemiology in Sudan. Transmission in Africa is partly zoonotic, as suggested by a high rate of infection in game wardens in Dinder Park, Sudan, which is otherwise uninhabited. However, the VL epidemic cycle in Sudanese villages is 8–10 years, and PKDL seems to be a reservoir of infection [10]. In Gedaref and Central State, Sudan, PKDL was prevalent without VL in 1990 [11,12], then in 1991, there was an upsurge in VL cases with an incidence of 38/1000 cases in one endemic village [13]. More than half of VL patients developed PKDL; in Sudan, PKDL follows VL in 56% to 62% of cases, 10% of PKDL cases present without a history of VL, and 15% of cases present at same time as VL (para-KDL). Patients typically present with PKDL within 6 months of VL treatment, sometimes as soon as 2 weeks, but all within 13 months after VL. The most affected age group is 4–8 years. Possible risk factors for developing PKDL include previous VL treatment (duration, drug), young age, malnutrition, human immunodeficiency virus (HIV) infection, genetic factors, and parasite strain [14]. The treatment given for VL may be an important factor; PKDL was found in 69% of VL cases after incomplete SSG treatment. Highly effective VL treatment may give rise to fewer PKDL cases (e.g., PKDL developed in 11% of patients with SSG monotherapy and 9% of patients with paromomycin [PM] monotherapy, but in only 6% of patients with SSG and PM combination treatment). PKDL was found in 19% of patients after a long course with low-dose PM and in 4% of patients after a short course with high-dose PM, pointing to a more effective intensive short course treatment, rather than a longer, lower-dose treatment course. The clinical presentation of VL seems to have no influence, but the parasitological status does. One study demonstrated that after VL treatment, patients with a polymerase chain reaction (PCR)-negative tissue aspirate (lymph node or bone marrow) did not develop PKDL (n = 20), whereas in those who were PCR positive, 36% developed PKDL (and 23% relapsed); therefore, parasite clearance during treatment may be of influence. High levels of C-reactive protein and high skin levels of IL-10 during active VL were linked to increased risk of PKDL. Several genetic factors were implicated, including decreased function of interferon gamma receptor gene (*IFN*-*γR1*) seen in PKDL patients (not in VL) [15].

Prof. Asrat Hailu described epidemiology in Ethiopia. The prevalence of PKDL is low in Ethiopia compared with Sudan but is higher in HIV co-infected patients, where it is often seen in an atypical presentation. VL occurs primarily in the lowlands but is scattered throughout the country, although the main endemic areas are in the north close to the border with Sudan (60%) and secondary foci in the south. VL mainly affects children and young adults, particularly in the southern foci, and in North Ethiopia there is a higher burden, mainly in adult males due to the number of people migrating from highlands to the lowlands for seasonal work. There is also a high HIV co-infection rate in this population.

Surveillance conducted in Gondar with a wide catchment area indicated that from July to December 2011, 243 patients were screened for VL, 67 were treated for VL, 22 of these (32%) were HIV positive and there were 3 PKDL cases. From January to April 2012, 183 patients were screened for VL, 75 were diagnosed as VL cases and there were 2 PKDL cases. From 2006 to 2011 in Sheraro district, 209 VL and 3 PKDL cases were treated; in Sulhul Hospital, 242 were treated for VL, 4 of whom were co-infected with HIV, but no PKDL cases were reported. In 2011 in Arba Minch (South Ethiopia) in a series of 109 treated VL cases; none were co-infected with HIV, and no PKDL cases were seen. In the past 30 years, only 8 cases of PKDL, 2 of whom were HIV co-infected, have been reported in the south. Limited unpublished data indicate that parasites in northern and southern foci differ, with those in the south more similar to parasites found in India, whereas parasite isolates from northern foci are more variable and similar to those in Eastern Sudan. The clinical relevance of this remains unclear.

Dr. Dia El Naiem provided a global entomology review. Of parasitic diseases, the transmission of *Leishmania* is probably the most complex, with a large diversity of parasites, vectors, and hosts. In the Mediterranean, old world *L*. *infantum* is transmitted by *Laroussius* species. In South Asia, *L*. *donovani* anthroponotic transmission is dictated by the high endophilic and anthropophagic behavior of *Phlebotomus argentipes*. The role of cattle is not yet well understood: cattle act as a zooprophylactic, but also increase the sandfly density. In the southern parts of East Africa, the vectors are *P*. *martini* and *P*. *celiae*; they reside close to each other, and their presence is related to termite mounds. In the Sudan, *P*. *orientalis* is the vector and resides in Acacia and Balanites forests. *P*. *rodhaini* is a newly identified vector. Even in non-endemic areas in the northern parts of East Africa, approximately 5% of sandflies are infected with *Leishmania*, both within villages and 40 km outside of villages, a finding that indicates a zoonotic reservoir. Infected mongooses and dogs have been found; jackals could play a role but are difficult to catch.

Experiments on the infectivity of PKDL to sandflies are few. In pioneering experiments in India, sandflies were held close to lesions in test tubes. In more recent work [16], conclusive experiments were performed using a hand with PKDL lesions inserted in a cage with sandflies, which more closely mimics real conditions. In Sudan, experiments have been performed by exposing nodular PKDL to wild populations of *P*. *orientalis*[17,18]. Large proportions of sandflies became infected. All these studies, however, were based on an extremely small number of samples and need to be repeated on a larger scale.

The main objectives of future experiments should be reliable estimates of the probability of infection and infectiousness when sandflies feed on different forms of PKDL versus clinical and subclinical VL, determination of how long a PKDL case remains infectious for sandflies, and determination of which types of PKDL lesions are most infective. Research in the Indian subcontinent showed that the parasite burden is higher in nodular and papular forms of PKDL than in the macular form [19]. Both laboratory-reared and wild populations of sandflies may be needed. When using colonized sandflies, exposure should be both to PKDL lesions as well as the whole body (using a large sandfly-proof bednet). This design should provide data on the rate that sandflies become infected as well as those that become infective.

#### Discussion of epidemiology

•Approximately 20% of PKDL cases in Rajendra Memorial Research Institute (RMRI), Patna, Bihar State, had no history of VL. For PKDL, longer treatment courses and higher doses are needed than for VL. PKDL cases occurred after all known VL treatments; indeed, out of 10,000 patients treated with AmBisome® 20 mg/kg in India by Médecins Sans Frontières (MSF), 0.9% developed PKDL (based on passive surveillance among those who reported to the clinic).

•The number of VL cases in Bangladesh is decreasing, and fewer districts are affected. There is a clear link between the number of VL and PKDL cases in active case finding around recent VL cases. Since 2008, hyper, moderate, and low endemic areas have been identified, and patients are mapped in villages. Patients are followed for 6 months after VL treatment to establish definite cure and PKDL rates. So far, few cases of PKDL have been recorded after adequately treated VL episodes. However, follow up should continue over a number of years to establish true PKDL rates.

•Ethical issue in PKDL: there is no standard guideline for diagnosis and treatment for PKDL, but because PKDL is not a life-threatening disease, treatments should not be long and toxic.

•Dr. Salotra has developed a method of using rK39 rapid diagnostic tests on slit skin aspirates, which could be used as an initial screening of samples before confirmation with PCR.

•A current VL epidemic with 25,000 patients in South Sudan has so far led to a mortality rate of 5%, which is much lower than previous rates due to increased access to care.

•In Libo Kemkem, Ethiopia, there was a high incidence of PKDL following a VL epidemic that occurred mainly in children; this is unusual for Ethiopia.

•Mathematical models can be based on infectivity experiments but will be very difficult to extrapolate. Large-scale studies cannot be performed using xenodiagnosis – biomarkers of infectivity of sandflies are needed. In South America, modeling suggested that dogs, and not foxes, are the reservoir hosts for VL.

•Many questions can only be answered through modeling: Assuming one case of PKDL in a village, how many sandflies feed on this case? How long do they remain infective? Can one PKDL case cause an epidemic, or is it of negligible impact?

•Differences between northern and southern Ethiopia include poor response to treatment and differences in parasites, but there are also host differences; the north has more malnutrition and high HIV co-infection rates, whereas in the south there are microfoci of VL, and people generally are better nourished. Differences overall are likely to be multifactorial.

•Infectivity work has mainly focused on nodular forms, but in Bangladesh the majority of the cases are macular or papular. Can these patients contribute to transmission? Prof. Benazir did not detect parasites in smears of macular lesions. In the past, there was more nodular PKDL in Bangladesh. Does the pattern change over time? In Bangladesh, some infected dogs and jackals were found, which raises questions about anthroponotic transmission.

•What is the probability of cattle acting as a reservoir for transmission of *Leishmania* by sandflies? Dr. Dia El Naiem assumes that cattle have strong complement fixation capacity, which would quickly kill the parasite.

•An alternative for xenodiagnosis experiments on humans is to feed sandflies with fresh blood through a chicken skin membrane.

•In a VL treatment study in Ethiopia carried out in 1998–1999, PKDL rates were 13.3% in non-co-infected VL cases and 27% in HIV co-infected VL cases; it is unclear whether these cases were from northern or southern Ethiopia.

•PKDL may not be an appropriate name because it often occurs without a history of VL.

### Session 2 Diagnosis

Presentation and discussion sessions on PKDL diagnosis were chaired by Prof. Asrat Hailu and Dr. Emily Adams.

#### Asia

Prof. Suman Rijal described PKDL diagnosis in Nepal. Diagnosis is performed in tertiary hospitals by dermatologists. Case detection is passive. Diagnosis is made by confirming a suspect case of PKDL with positive rK39, PCR, or demonstration of the parasite on punch biopsy. Between 1998 and 2011, 106 PKDL cases were diagnosed at the BPKIHS in Nepal: 64 were males (60.2%), mean age was 27.5 years (range 6 to 60 years), almost all cases had a past history of VL, with a mean time of appearance of lesions after the VL episode of 27 months (range 2 to 60 months). VL was mainly treated with SSG in almost all cases, but with inadequate doses in 2/3 of cases. Two cases presented with PKDL after miltefosine. No cases were seen after treatment with amphotericin B. Presentation was macular, papular, and nodular, with lesions on the face, body, and extremities.

Of 106 cases, parasites were found in biopsies in 43 cases (40.5%) by microscopy but none were found in macular lesions. Histopathology in macules showed dermal infiltrate consisting of lymphocytes, histiocytes, and plasma cells; in plaques a dense cellular infiltrate in the dermis consisting of lymphocytes, plasma cells, and histiocytes with abundant plasma cells. In nodules, granulomas were found extending down to the subcutis and formed by macrophages, giant cells, and infiltration consisted of lymphocytes and plasma cells. Better tools are needed as skin slit microscopy is specific but not sensitive.

Prof. Yoshitsugu Matsumoto and Dr. Eisei Noiri described VL diagnosis in Bangladesh. A joint project between the University of Tokyo (UT) and ICDDR,B was presented, which aims to develop diagnostic technologies suitable for use in the field. Loop-mediated isothermal amplification (LAMP), biomarker urine tests, and immunofluorescence are being tested for diverse purposes:

•Detection of VL: abcDAT and rKRP42, which remain positive after disease.

•Evaluation of side effects, progression of cure, and resistance to treatment: LAMP and L-fatty-acid-binding protein (L-FABP; in urine), which become negative as the patient is cured.

L-FABP has been identified as a useful biomarker that can indicate acute kidney injury and is, although nonspecific, indicative of acute VL. A rapid test (dipstick) for urine is being developed using enzyme-linked immunosorbent assay (ELISA), rKRP42, and L-FABP that will diagnose VL, reflect drug resistance, and assess renal injury as well as multiple organ failure.

A test used to confirm PKDL diagnosis could be based on the same concept [20].

Dr. Poonam Salotra described diagnosis in India. PKDL in India can affect any age group, and accurate diagnosis is a challenge because there are few parasites in the lesions and blood. Parasitological confirmation is usually done in a hospital setting. Histopathology may show *L*. *donovani* (LD) bodies, which may not be seen in pathology samples; typically, there is an infiltration of lymphocytes, histiocytes, and plasma cells, but this level of histopathology requires experienced technicians and is not very sensitive. Results of studies conducted with antibody-based tests, such as rK39 and DAT are specific for VL/PKDL, but remain positive in patients even 10 years after initial infection [21] and are therefore not able to discriminate between VL and PKDL.

DNA-based tests are both sensitive and specific and can be used on skin, blood, and bone marrow. These methods can be used as a confirmatory test in hospital testing. Species-specific PCR on slit skin smear demonstrated a sensitivity of 93.8%. First-round PCR on slit skin smear gave a sensitivity of 63%, which increased to 93% with nested PCR. kDNA has the best sensitivity of 93% on slit skin smears and 94% on skin biopsy [22].

In a study of 62 PKDL slit skin tissue samples using quantitative PCR (qPCR), all were positive. Nodular cases had a higher parasite load than macules. Using a slit skin smear with part of the sample tested using rK39 rapid diagnostic tests and part using PCR demonstrated a significant correlation between the rK39 test and PCR, meaning that rK39 rapid diagnostic tests in skin slits smears could be used as an initial diagnostic screening for PKDL [23]. Also, a correlation has been shown between interleukin-10 (IL-10) levels and parasite load.

Another technique, LAMP, is easy to operate, has a quick reaction time, uses naked eye detection, and can be used on blood samples or tissue including skin smear samples from PKDL patients. In VL patients, 53 of 55 blood samples were positive, giving a sensitivity of 96% and a specificity of 97%; in tissue samples from nodular and macular lesions from PKDL patients 60 of 62 samples were positive, giving a sensitivity of 97% and a specificity of 100%. However, this test needs further large-scale evaluation in the field [24].

#### Africa

Dr. A Mudawi Musa was unable to attend the meeting. Prof. Ed Zijlstra presented on behalf of Dr. Musa and described diagnosis in Sudan.

PKDL can be diagnosed either clinically or parasitologically. The clinical diagnosis is based on the following:

•The appearance of the rash: macular (which does not follow the same distribution as other forms), papular (which may be micropapular: measles-like rash), or nodular.

•The distribution of the rash: in Sudan, the rash usually starts around the mouth, then spreads to the nose and cheeks and finally to other parts of the face and body.

•Temporal relation to VL treatment, although approximately 10% of patients who develop PKDL do not have a history of VL. In Sudan, PKDL usually develops within 6 months and almost all cases present within 13 months after VL; young children are most commonly affected.

•Possible differential diagnoses with PKDL include the following:

○ Leprosy, but leprosy is associated with anaesthetic lesions and enlarged nerves and is not more prevalent in sun-exposed parts of the body.

○ Vitiligo is characterized by depigmentation in contrast to the hypopigmentation seen in PKDL.

○ Miliaria (prickly heat) is characterized by tiny papules in small children wrapped in clothes despite hot weather.

○ Acne, typically in adolescents, which can also present with comedones.

○ Measles, where the rash may appear the same, but is associated with other clinical symptoms, such as conjunctivitis, Koplik’s spots, and otitis media as a complication.

Clinical diagnosis is possible, but sometimes requires an experienced physician. Confirmatory parasitological diagnoses, although difficult, are possible using the following techniques: (1) staining of slit skin smears or skin biopsies for direct microscopy and (2) use of special techniques including monoclonal antibodies or PCR. It should be noted that serology is of limited use because it cannot differentiate between VL and PKDL, and patients remain serologically positive for long periods of time after VL [25].

#### Discussion on PKDL diagnosis

•PCR diagnosis in Bangladesh: Sensitivity in blood was 40%, but most patients have macular lesions, suggesting a low parasite load. Slit skin smear positivity was 46% and, when combined, the sensitivity increased to approximately 60%. However, PCR methods are not feasible in the field in Bangladesh.

For initial diagnostic screening, slit skin smear samples should be obtained, transferred into buffer, and part of the sample may be used for rK39 rapid diagnostic tests in the field and part is sent to a laboratory for PCR analysis.

•How feasible is it to decentralize LAMP for use in the field? The procedure is relatively simple but not ready to be decentralized. qPCR may be more practical but still requires RNA and DNA preparation, which may be difficult to implement outside of a hospital setting.

•Are PKDL and VL *Leishmania* parasite strains different? To date this has not been seen by Prof. Hailu, Dr. Salotra, or Prof. Matsomoto [26].

•There is no user-friendly consistently reliable diagnostic test for PKDL, which is problematic because current treatments are either long and toxic or expensive.

•There are no good reference standards available to evaluate PKDL diagnostic tests.

### Session 3 Pathogenesis

Presentation and discussion sessions on pathogenesis of PKDL were chaired by Prof. Farrokh Modabber and Dr. Pradeep Das.

#### Asia

Dr. Poonam Salotra described immunology in India. Ideally, one would evaluate the immune response in VL patients and then follow them to see whether they develop PKDL. This is not feasible in India because of the interval between VL infection and development of PKDL. However, patients who were evaluated pre- and post-treatment for PKDL demonstrated increased cell-mediated immunity. Elevated levels of cytokines including IFN-γ, IL-6, and IL-10 were seen in blood samples from active VL compared with controls, whereas tumor necrosis factor (TNF)-α was elevated in PKDL. In skin tissue samples, all cytokines measured (IFN-γ, TNF-α, IL-6, IL-10, transforming growth factor [TGF]-β) were elevated in PKDL; IFN-γR1 levels were low. After treatment, all cytokine levels were reduced and IFN-γR1 levels increased.

Analysis of host immunodeterminants in localized infected tissue of PKDL patients using cDNA implicated IL-17, chemokines, and CD25 and key molecules of host signaling pathways (FasL, Fas, interferon regulatory factor [IRF]-1, protein-trypsin kinases).

In PKDL, the presence of peripheral CD8+ CD28 T regulatory cells suggests their role in disease pathology. The presence of T regulatory cells in PKDL patients and controls was evaluated using specific markers (CD25, IL-10, Foxp3): they were up-regulated in tissue samples prior to treatment and declined after treatment. T helper (Th) 17 markers (IL-17, IL-23) were also up-regulated in PKDL tissue and reduced after treatment. Production of IL-17 and IL-23 upon stimulation of peripheral blood mononuclear cells (PBMCs) with total soluble *Leishmania* antigen (TSLA) indicates a potential role for T regulatory cells in parasite persistence, possibly via secretion of IL-10. PBMCs of PKDL patients produce TNF-α and NO upon stimulation with IL-17, indicating a role of IL-17 in PKDL pathogenesis.

Investigations on T regulatory cells and Th17 responses revealed a paradoxical gene expression signature combining markers associated with inhibition of the immune response (T regs) and the pro-inflammatory response (IL-17) in PKDL. It was proposed that during PKDL, the parasite induces the production of T regulatory cells possibly by secreting IL-10, which counteracts the pro-inflammatory cytokines (IL-17, TNF-α, and nitric oxide [NO]) and promotes parasite persistence.

#### Africa

Prof. Ahmed M. El-Hassan was unable to attend the meeting. Prof. Ed Zijlstra presented on behalf of Dr. El-Hassan and described pathology, parasites, and genetics of PKDL. PKDL occurs in the context of immune (re) constitution. This is demonstrated by the proportion of leishmanin skin test (LST) positives during various stages of infection: all patients are LST negative in VL, 11% are positive in para KDL, 27% are positive in PKDL, and 80% are positive in cured VL. Serology is positive in 90% to 100% of VL patients and in 80% to 90% of PKDL patients. The immune response is predominantly Th2 in VL and a mixed Th1/Th2 response in PKDL.

In a study of 20 VL patients followed for 6 to 24 months, parasites were seen in the skin during VL in both those who did and did not develop PKDL. This means that parasites do not ‘take refuge’ in the skin after VL, as had previously been hypothesized. In patients who developed PKDL, IL-10 levels in the skin and after PBMC stimulation were higher at initial diagnosis of VL; however, no difference in IL-10 production was observed at later points in time [27]. In both groups, IFN-γ production and PBMC proliferation were absent.

Of 134 untreated non-severe Sudanese PKDL patients, 84% self-healed within 15 months. In contrast to those with persisting PKDL, most self-healers developed a positive LST test and had low titers in the DAT.

The severity of PKDL in Africa seems related to exposure to ultraviolet (UV) light and immaturity of the immune system, which reflects the more severe forms typically seen in small children, who often spend more time outside; PKDL incidence after VL differs greatly: 56% to 62% in Sudan, 14% in Ethiopia, and 0.05% to 30% in Kenya. Parasite strain testing in Africa rendered no particular parasite isolated in PKDL; all are caused by *L*. *donovani* parasites that are heterogeneous and different from those found in Europe and South Asia. Genetic factors have also been described: decreased function of *IFN*-*γR1* gene was observed in PKDL (and not in VL), and polymorphism in the Th2 cytokine gene was found to be linked to PKDL.

#### PKDL in relation to treatment

Dr. Epco Hasker described PKDL in relation to treatment. Because the incidence of PKDL has declined concomitantly with the increase in use of amphotericin B to treat VL, it has been suggested that antimonials could play a role in PKDL development. Gupta *et al*. described a peroxisomal disorder after successful treatment for VL with antimonials, which could be one possible reason that PKDL develops [28].

Saha *et al*. described an increase in IL-10 and TGF-β in some antimony-treated individuals, an elevation that is also seen in patients with PKDL, which may suggest a role of these cytokines in reactivation of the disease in the form of PKDL [29]. This cytokine response is not seen with amphotericin B treatment, possibly reflecting its better therapeutic activity and probable role in the recent decline in PKDL.

In fact, the tendency to develop PKDL may depend on a number of factors including the individual’s response to SSG treatment as a result of parasite load, genetic predisposition, environmentally acquired traits, or a combination of such factors including inadequate treatment, meaning that patients who receive adequate treatment may not develop PKDL.

Published studies in Sudan and Bangladesh demonstrate that a variable number of patients treated with SSG develop PKDL; the general trend over time is that the number of patients treated with SSG who subsequently develop PKDL increases with time since treatment.

Overall, there is a large bias because SSG has been used much more widely and routinely than other drugs and it is generally not known whether treatments are fully completed. PKDL has also been reported after treatment with amphotericin B, miltefosine, and PM; but thus far, a lower number of patients seem to develop PKDL following these treatments.

#### Discussion of pathogenesis

•There may not be a true difference in PKDL incidence following VL between Sudan and Ethiopia, due to a reporting bias: indeed, only severe cases requiring treatment are officially reported in Ethiopia (only 50% of cases are severe [grade 2 or 3]).

•There is a strong immunologic component to PKDL, but underlying genetic factors have also been identified.

•The dose of the antigen can direct the immune response and can also influence it.

•Isoenzyme analysis will demonstrate the evolution of the parasite, but how this relates clinically is not known.

•Treatment is important, but healing also involves an immunologic response. Moreover, patients could be ‘cured’ (i.e., parasite clearance is reached), but it takes a longer time for the skin lesions to heal, particularly in severe cases. The question is how to shift the immune response, and whether clinically cured lesions still contain parasites.

•A genetic factor is likely because PKDL in Bangladesh tends to occur within families.

•*In vitro* drug sensitivity testing showed a difference in sensitivity of VL and PKDL isolates with miltefosine and parasites from PKDL patients being more sensitive to miltefosine, although the differences were only 3- to 4-fold.

•There is a difference in the systemic immune response (blood) versus local immune response (skin biopsies).

•A shift from a Th_2_ response in VL to a mixed Th_1_/Th_2_ response in PKDL is most important. What type of immunomodulator could be used?

•Is the severity of VL related to PKDL? A suggested experiment is to follow 1,000 VL patients, including studying their immune profile and the rate of PKDL.

•OWH will start a 4-year multi-site systematic follow-up study of 1,000 VL patients who have had different treatments to detect the PKDL rate for each drug.

•Is the T cell response in the skin related to PKDL? LST positivity is often seen in PKDL.

### Session 4 Clinical presentation

Presentation and discussion sessions on clinical presentation of PKDL were chaired by Dr. Jorge Alvar and Dr. Dinesh Mondal.

#### Asia

Prof. V. Ramesh described clinical presentation in India. In the Indian context, as in Sudan, PKDL is considered a sequel to VL, with parasites in the skin but generally not in the viscera. Leishmanioma has not been described in the Indian context. A total of 227 PKDL cases were reported at a Delhi hospital between 1996–2011; patients were predominantly from Bihar and the surrounding states, but migrated to Delhi for work where they were seen in tertiary hospital settings. PKDL has been reported in other parts of India, but all patients were from endemic regions, Bihar in particular. Clinical presentation included hypo-pigmented macular, papular, and nodular lesions [30]. Approximately 20% of patients had no history of VL, but came from an endemic area and had confirmed parasitological diagnosis of PKDL.

The most common presentation in India is a mixed presentation of macules, papules, and nodules that constitute more severe cases that do not heal spontaneously [31]. The face is usually most severely affected, but lip borders, inner cheeks, genitals, tongue, and hard and soft palate can also be affected; mucosal lesions in PKDL are seldom seen, with only one case reported in the highly endemic area of Bihar at the height of an epidemic.

Prof. Ridwanur Rahman described the clinical presentation of PKDL and VL in Bangladesh. Because VL and PKDL were not reported separately until last year, the burden is unclear. Eighty-nine cases of PKDL were identified and studied: mean age was 22 years with a male and female ratio of 1.1. Most patients were very poor, with a per capita income of less than 2 USD/day. The majority of them (~70%) were students/up to secondary school, and approximately 85% had no knowledge about agent, transmission, and treatment of the disease. Herbal treatment or medicinal ointment was used by about 68% of patients. The mean duration of lesions was 1–96 months, and time since treatment was 8–96 months. Fifteen percent of cases had no prior history of VL. There was a family history of VL in 44% of cases. Macular lesions occurred in 68% of patients, maculo-papular lesions in 27%, and maculo-papulo-nodular lesions in 6%. Ninety-three percent were rK39 positive, and 68% were skin smear positive. In 3 cases, rK39 was negative, but slit skin smear and PCR was positive. In macular lesions only, LD bodies could be demonstrated in 45%; all nodular and papular cases were slit skin smear positive. Treatment consists of SSG for 20 days, with an interval of 10 days, repeated 5 times, after which lesions generally disappear 6 months to 1 year after completion of the SSG courses [32,33].

In the SKKRC in Bangladesh, VL is treated by conventional amphotericin B for 15 days, lipid amphotericin B (produced in India; requires sonication before use) for 5 days, miltefosine for 28 days or SSG for 30 days. PKDL is treated with miltefosine or liposomal amphotericin B. PKDL developed with no previous treatment for VL and was seen after various VL treatments: miltefosine, PM, SSG, AmBisome®+miltefosine and PM+miltefosine, indicating that occurrence is probably not drug-specific. The frequency of development of PKDL should be considered an endpoint in clinical trials for VL.

#### Africa

Dr. A Mudawi Musa was unable to attend the meeting. Prof. Ed Zijlstra presented on behalf of Dr. Musa and described PKDL clinical presentation in Sudan. A variety of clinical slides were shown to illustrate differential diagnoses. Macular, papular, or nodular rash may be seen alone or in combinations. The severity is usually described in 3 grades. PKDL can be self-healing, responsive or not to standard treatment. PKDL leaves no residual lesions, except patients with chronic PKDL, in whom the skin may become fibrotic.

#### PKDL in immunocompromised patients

Prof. Ed Zijlstra described treatment of immunocompromised patients, who are primarily HIV co-infected patients. In these cases, both HIV and VL need to be treated (*i*.*e*., the patient needs to be initiated on highly active anti-retroviral therapy [HAART] and anti-leishmanial therapy to reduce both the viral load and the number of parasites). This combined treatment should allow the patient’s CD4 count to improve and achieve a reduction in VL symptoms and possibly achieve initial parasitological cure.

PKDL is more common in HIV-positive patients, often in an atypical evolution and presentation, such as atypical large nodular lesions, unlike normal PKDL patients. The face may or may not be affected. Other differences include a higher parasite load and although the parasite is usually *L*. *donovani*, PKDL in co-infected patients caused by *L*. *chagasi*/*L*. *infantum* has been reported. Para KDL is more common and more severe in immunocompromied patients.

The terminology in the literature used to describe the atypical presentation seen in co-infected HIV patients is varied and inconsistent, but can generally be classified as disseminated cutaneous lesions (CL) without VL, disseminated CL preceding VL, disseminated CL and VL concurrently, and disseminated CL following VL. A proposal for consistent terminology could be ‘pre kala-azar dermal leishmaniasis,’ ‘para kala-azar dermal leishmaniasis,’ and ‘post kala-azar dermal leishmaniasis.’

#### Discussion of clinical presentation

•With regard to the natural history of the disease, 80% of cases in Bihar are macular, and approximately 80% of patients subsequently develop nodular and papular lesions.

•In Bangladesh the differential diagnosis of PKDL includes chronic arsenic poisoning (due to contamination of water in some areas of India and Bangladesh), but the epidemic areas do not overlap.

•In Bangladesh blurred vision is seen in some PKDL patients (linked to the affection of the retina previously mentioned), which can lead to blindness if not appropriately treated.

•In Asian PKDL, macular lesions are common; because grading of severity is difficult in these lesion types, it is not typically done in Asia. However, it could be a useful tool to assess treatment response.

•Macular lesions are a more common presentation in children, and nodular and papular lesions are more common in adults in Asia.

•PCR performed on skin outside of lesions was negative, so only the lesions may be infective in PKDL.

### Session 5 Treatment

Presentation and discussion sessions on treatment of PKDL were chaired by Dr. Philippe Desjeux.

#### Asia

Prof. V Ramesh described treatment in India. In India, all patients are treated, irrespective of the duration of PKDL. The current recommended treatment remains the same: SSG, as it has been for the past 50 years, and in the words of Prof. Marsden “this is nothing to be proud of” given the toxicities associated with the use of antimonials. Antimonials are given for up to 120 days at the same dose as for VL, i.e. 20 mg/kg; however, parasites can no longer be detected microscopically in lesions after 2 months of treatment. Due to its length and associated toxicities, patients have difficulty tolerating the treatment. There is a high dropout rate because patients, or their parents in the case of infected children, need to continue working, which is difficult if they have to attend a clinic for daily injections. Immunochemotherapy is an interesting option; SSG+Mycobacterium w. vaccine and SSG+rifampicin were compared, and a minimal difference was seen between both treatments [34].

High doses of amphotericin B are needed to treat PKDL; total doses of up to 4.5 g are given as 1–1.5 mg/kg/day, compared to doses of up to 1 to 2 g for treatment of VL. Another option is miltefosine, which has the advantage of being an oral drug. A small number of patients have been treated at doses of 50 mg three times per day (tid) for 2 months and 50 mg twice per day (bid) for 3 months in patients who were unable to tolerate the tid dosing regimen. This treatment reduced the severity of lesions by the end of treatment (at which point parasites were absent), but it took an additional 4–6 months for lesions to heal completely. In some cases, the lesions subsided but the glandular swelling remained [35,36].

Monotherapy for PKDL seems to require at least 3–4 times higher doses than for VL, and even this may not be sufficient. Could combination therapy reduce the length of treatment? In one patient, amphotericin B 1 g total dose combined with miltefosine 50 mg tid for 40 days resulted in cure. It is unknown whether this dose could be reduced further.

Dr. Dinesh Mondal described treatment in Bangladesh. In a literature review, only 31 papers on treatment of PKDL could be found (key words: post-kala-azar dermal leishmaniasis+treatment), and all were studies with very small numbers of patients. The following drugs have been used to treat PKDL: SSG, rifampicin, ketoconazole, nystatin, terbinafine, itraconazole, allopurinol, amphotericin B deoxycholate, liposomal amphotericin B, miltefosine, and immunotherapy. Furthermore it was documented that:

•20 mg/kg SSG regimen for 120 days had the highest cure rate compared with 10 mg/kg and 15 mg/kg (India)

•No major advantage was obtained using allopurinol, rifampicin, or Mw vaccine (heat-killed Mycobacterium) along with SSG versus SSG alone (India)

•SSG+Bacille Calmette-Guérin (BCG)+Alum-precipitated autoclaved *L*. *major* regimen proved safe and had a higher cure rate (87%) versus 53% with SSG alone (Sudan)

•Amphotericin B deoxycholate at a dose of 1 mg/kg on days 1–20, 21–40, and 61–80 cured 11/11 (100%) versus 7/11 (63%) with SSG at a dose of 20 mg/kg for 20 days (4 to 6 courses) (India)

A total of 30 PKDL patients were reported to be successfully treated with miltefosine (all Indian), and 11 cases were reported as cured with AmBisome®, at a dose of 2.5 mg/kg for 20 days (10 cases, Sudan) and AmBisome at a dose of 3 mg/kg for a total of 15 days (one case). The duration of lesions showed a positive correlation with the duration of treatment necessary: macular lesions heal more slowly than the papular lesions. Other tested drugs were found non-effective. Efficacy studies for miltefosine, amphotericin B, and AmBisome® for treatment of PKDL are highly desired, and the role of immunotherapy to prevent development of PKDL should be systematically studied. Biomarkers (e.g., IL-10) should be found that will predict development of PKDL and provide a test of cure. Based on past and current findings, a guideline for treatment of PKDL is urgently needed.

#### Africa

Dr. A Mudawi Musa was unable to attend the meeting. Prof. Ed Zijlstra presented on behalf of Dr. Musa and described treatment in Africa. Current unresolved issues are as follows: who needs treatment (from the clinical and epidemiological perspective), which drug regimen is the best, and how to monitor outcome (clinical, immunological, or parasitological cure), which is important in the conduct of clinical trials as well as for routine patient care to avoid overtreating patients with expensive and/or toxic drugs. Current practice is treatment of all severe grade 2 and grade 3 patients with SSG 20 mg/kg with the length of treatment (60–120 days) based on clinical monitoring. AmBisome®treatment for PKDL has been tried in Sudan in 11 patients who did not respond to SSG. AmBisome® 2.5 mg/kg × 20 days was given; 83% healed, with macular lesions being slower to heal than papular ones, and a longer duration of lesions meant a slower healing process [37]. Suggested new approaches are shorter courses of AmBisome®, or combinations such as SSG+PM (already in use by MSF for PKDL), miltefosine, and immunotherapy or immunochemotherapy. The latter was tried in chronic PKDL (>6 months): an autoclaved *L*. *major* /alum vaccine was given to 30 patients. SSG+vaccine+BCG cured 87% of patients versus 53% with SSG alone [38]. Vaccine efficacy was 71% (95% confidence interval for risk ratio, 0.7-1.16), and IFN-γ production was augmented and LST converted in the vaccine group, which indicates a desired shift to a Th1 response. Currently, a vaccine developed by the Infectious Disease Research Institute (IDRI), Seattle, Washington, is being tested (phase 1). It consists of a recombinant three-antigen *Leishmania* polyprotein (Leish-F2 with adjuvant monophosphoryl lipid A-stable emulsion [MPL-SE]) and will be tested in combination with SSG. Immunochemotherapy is a long-term goal; new immunomodulators are not expected to enter clinical studies before 2014. Better drug therapy (short course, ambulatory, inexpensive) should be developed in the shorter term, such as perhaps 1–2 doses of AmBisome®+ miltefosine. Another research priority is the prevention of PKDL via investigation into which VL treatments result in the lowest PKDL rates.

#### Médecins sans Frontières (MSF) field experience

Dr. Koert Ritmeijer described MSF field experience with treatment. In Africa, diagnosis is clinical using the following severity scale:

•Grade 1: Sparse macular, papular, or nodular rash of the face around the mouth with or without lesions on the upper chest and upper arms.

•Grade 2: Dense maculopapular or nodular rash covering most of the face and extending on the chest, back, and upper arms and legs. If extensive or black nodules, it is grade 2 (severe).

•Grade 3: Dense maculopapular rash, covering most of the body, including hands and feet. In grade 3, crusting, ulcers, scaling, and spreading to the mucosa of the lip and the palate occurs.

Only severe grade 2 and grade 3 PKDL are treated.

In Sudan, treatment at base sites (under physician supervision) is with a combination of SSG (20 mg/kg per day intramuscularly) for up to 60 days, plus PM (15 mg [11 mg base] per kg per day intramuscularly) for 17 days. At outreach sites (no permanent physician supervision) SSG 20 mg/kg per day intramuscularly for 30–60 days is used. Treatment lasts until lesions are definitely improving (not until lesions have disappeared). Thus far, 422 PKDL patients have been treated, all of whom self-reported (no active case finding); 65% had mucosal involvement, with consequential visual impairment sometimes present. With SSG + PM, significantly fewer treatment days were needed to achieve cure versus SSG alone (34 *vs*. 41 days, p < 0.001); 10% needed >60 days of therapy. With SSG+PM, fewer patients defaulted than with SSG alone (9% *vs*.3%). Mortality with SSG alone was 0.9%, and no patient died with SSG+PM (0%). Other observations: The median interval between treatment for VL and admission for PKDL was 84 days, and the median interval between completion of VL treatment and onset of PKDL was 26 days. Median age was 5 years, mean age was 10 years, and 49% of cases were female. Young age is a risk factor for developing severe PKDL.

In Ethiopia, the incidence of PKDL is unknown due to the migrant character of VL patients and the lack of systematic follow up. In a clinical trial with a 6-month follow up, the incidence of severe PKDL was 13.3% in HIV-negative and 27.3% in HIV-positive patients [39]. HIV is clearly a risk factor for PKDL. Treatment is with SSG (20 mg/kg/day intramuscularly) for 30–60 days. In total, 141 PKDL patients were treated with a mean of 38 SSG injections. Two HIV-positive PKDL patients were successfully treated with miltefosine (100 mg × 28 days) [40].

In Bangladesh (Fulbaria, Mymensingh district), in MSF’s VL clinic, VL and PKDL are treated with AmBisome. Ninety-six percent of PKDL patients present with macular lesions, mostly non-severe. Case detection is active, and all detected patients receive treatment in the context of VL elimination. Treatment is with AmBisome® 30 mg/kg total dose (5 mg/kg twice weekly for 3 weeks). Patients are followed up for a minimum of 12 months. Over 1000 patients have been treated thus far; median age was 18 years. It took a median of 3 years for PKDL symptoms to develop after VL, and 7.5% developed PKDL without a history of VL.

Efficacy of treatment was evaluated by scoring of lesions and medical photographs; based on the drastic improvement of lesions between 1 and 3 months in most patients, it is thought that parasite clearance takes place during that time and that further improvement of lesions is an ongoing slow repigmentation process. In 90% of patients, a response to treatment was observed. Side effects were minimal, but 2% showed possible or confirmed rhabdomyolysis. This was unexpected and probably due to contextual factors; treatment was stopped until improved routine monitoring for any clinical signs of hypokalaemia, routine serum potassium, serum creatinine, and urine pH before, during, and after treatment, and potassium supplementation for all patients could be implemented.

Treatment for VL is with AmBisome® (15 mg/kg). Ten percent of VL patients treated in 2010 for VL (44/442) have developed PKDL (18–24 months post-treatment).

In India (Bihar State, Hajipur, Vaishali district) since 2007, MSF treated over 10,000 VL patients; PKDL incidence is unknown (but an active retrospective follow up of 18 months for a patient cohort is in progress). Despite health education on PKDL, thus far only 15 of 6368 VL patients (0.2%) treated with 20 mg/kg AmBisome® have presented at the clinic with PKDL. It took a mean of 2 years for PKDL symptoms to develop after VL. A prospective study evaluating the safety and efficacy of AmBisome® in the treatment of PKDL patients is ongoing (in collaboration with RMRI). Forty-five patients with confirmed skin slit parasites were treated with AmBisome® 2.5 mg/kg for 20 days (2 or 3 courses). Parasitological evaluation of progress (slit skin smear) was carried out after each course. Preliminary outcome is as follows: all patients were parasite negative after 2 courses, and clinical cure was achieved in the majority after 3 courses. No serious side effects were observed. Costs of this treatment are prohibitive for general roll out.

#### Immune modulation outlook

Prof. Farrokh Modabber described the current outlook for immune modulation approaches to treatment. Leishmaniasis is an immunological disease, and sterile immunity may not be possible. One proposal is to use immunochemotherapy, in which a patient is treated with an anti-leishmanial drug to reduce the parasite load and then with immunotherapy to promote an immune response. There are no animal models for PKDL, and the immune control mechanisms seem to be different in PKDL compared with VL. A number of studies have evaluated immunochemotherapy in PKDL patients, including autoclaved *L. major*/Alum vaccine+BCG in combination with SSG, which resulted in all patients being cured (15/15) compared to 6/15 patients cured with 2 patients relapsing when treated with SSG alone. There was no follow-up trial. BCG is inexpensive but takes 6 months to result in a cure. BCG is a T cell adjuvant but is very weak, and newer adjuvants that promote a T cell response have been developed. Other vaccines in development include the following [41]:

•MPL-squalene – but squalene promotes a Th2 immune response, which may not be ideal for PKDL.

•KSAC + glucopyranosyl lipid adjuvant-stable emulsion (GLA-SE) and synthetic MPL, which is currently in phase 1 development with Infectious Disease Research Institute (IDRI).

•RAPSODI – developed from the canine vaccine Virbac that is protective in dogs and contains identified active peptides.

•LeishDNAvax – evaluated in animal models (mice and hamsters), and 5 antigens that were most highly conserved in *Leishmania* species were selected and incorporated into DNA constructs. Toxicology studies have been completed, and the next stage is phase 1 clinical trials.

Another possibility is the use of an immune modifier, and one possibility is CpG. These are species specific. A possible candidate has been developed by Daniela Verthelyi, at the Food and Drug Administration laboratories. In monkeys with cutaneous leishmaniasis, a single injection of CpG with no additional antigen showed a good immune response as defined by an increase in IFN-γ. One proposal following toxicity studies could be to use this immune modifier plus chemotherapy with either AmBisome or miltefosine.

#### Discussion of treatment

•In Nepal, SSG is no longer used; PKDL patients have been treated with miltefosine for 12 weeks. Some patients relapsed after approximately 1 year.

•Do Indian /Bangladeshi patients who have mild lesions heal spontaneously? This seems rare but it is known to occur sporadically.

•A general concern was raised about the 10% rate of PKDL seen following treatment with 15 mg/kg AmBisome® in Bangladesh versus the 0.2% seen in India, where a higher dose of AmBisome® is used (21 mg/kg total dose). However, this difference is very likely due to the active case finding conducted in Bangladesh to identify PKDL cases. PKDL is more likely due to an immunologic component than to treatment or insufficient treatment.

•Although hypokalaemia leading to rhabdomyolysis was an unexpected side effect, AmBisome® as a useful drug for treatment of PKDL should not be discredited. Many patients probably have mild or moderate hypokalaemia during treatment, and only a few progress to rhabdomyolysis. Potassium supplementation should prevent this side effect.

•Treating mild PKDL in Sudan with SSG is not recommended because of its side effects that make this treatment unethical, particularly because PKDL will self-heal in many patients.

•In Sudan, PKDL relapses were seen, but PKDL progressing to VL was not seen.

•In Bangladesh, many patients did not achieve full cure of macular lesions after 12 months; these patients were followed further, and it was observed that the clearing of lesions continued after 12 months.

•How is cure of PKDL defined? A definition of cure is important; an ideal would be a simple parasitological endpoint that could occur well before repigmentation and resolution of lesions, which may take much longer.

•Concern was raised about the potential safety issues that could be associated with immune modifiers.

•Is there a nutrition factor involved in development of PKDL? Perhaps arginine (this is being investigated) or vitamin D deficiency (leading to low immune response)?

### Session 6 Control

Presentation and discussion sessions on control of PKDL were chaired by Prof. Be-Nazir Ahmed and Dr. Garib Das Thakur.

Dr. Be-Nazir described current control measures in Bangladesh, which include the following:

•Ensuring early diagnosis and treatment.

•Districts were classified as low (<1/10,000), medium (1-2/10,000), and high (>2/10,000) endemicity for VL, and teams were trained to do indoor residual spraying (IRS).

IRS (using deltamethrin) was initially piloted in the highly endemic Fulbaria district and has since been extended to 7 other highly endemic districts in Bangladesh.

•In parallel, active case finding has been carried out around VL and PKDL cases reported in the last years in districts classified as being of low endemicity. If new cases were identified, focal spraying was conducted, insecticide-treated bed nets (ITBN) provided, and fieldworkers trained to identify possible PKDL cases that are then referred for assessment by a physician.

•In the future, IRS is planned for medium endemic districts, where the camp approach will be used to detect patients [42-44].

Dr. Philippe Desjeux described the methods and strategies for PKDL control. He explained that PKDL cases deserve much more attention because PKDL is highly relevant in the context of VL elimination. Increased financial and political commitment are urgently needed to implement research recommendations in order to improve control strategies [45]. A key question in VL control is whether PKDL can contribute to transmission and cause outbreaks. There is historical evidence for this: in West Bengal, India, VL entered the Malda district in the North through migration from Bihar State. PKDL cases moved to a village in 24-Parganas district in the South where, consequently, VL started to occur in an isolated focus [16]. Other evidence has been generated via xenodiagnosis: of 400 specimens of laboratory-bred *P*. *argentipes*, 104 (26%) fed on PKDL nodular cases, and of 60 surviving sandflies, 32 (53%) became infected [16]. In real-life circumstances, it was observed in Sudan that nodular PKDL, in a boy, infected 25% of *P*. *orientalis* sandflies [17]. Via modeling, it was concluded that the presence of as few as 0.5% durably infectious (PKDL) patients during an epidemic may cause VL to become endemic [46].

Because PKDL is a chronic disease that has severe cosmetic stigma but is not fatal, patients generally do not demand treatment. PKDL in Africa was even seen as a favorable sign: “the disease has come out! The child will survive…” Costly, long, and toxic treatments with SSG also cause patients to refuse treatment: “I will better die than to receive 120 injections.”

Control of PKDL is only possible through a multifaceted approach, which needs (1) strengthening the surveillance system to evaluate the real incidence of PKDL and to reduce morbidity; (2) strengthening early detection and prompt treatment, including mapping; (3) promoting vector control through the use of long-lasting treated nets (LLTNs) if contribution to transmission is confirmed; (4) increasing community awareness, education activities, and promotion of health-seeking for VL and PKDL; (5) promoting adherence to treatment through shorter treatments and reimbursement of transportation costs for the patient; and (6) encouraging capacity building through training of health personnel at all levels and lab strengthening [45]. The recent publication of a WHO PKDL atlas, the development of a manual for healthcare workers, and WHO guidelines for PKDL management are a welcome development.

Recommended PKDL case detection is through a cluster approach: a mixed approach of active and passive case detection (based on digital mapping) around an index case, with suspected cases detected by community health workers or self-reported by patients, to be confirmed by physicians (referral system).

To assess the drug-specific incidence rates of PKDL after various VL treatments, PATH – OWH will soon initiate a PKDL follow-up project in Bangladesh: 1,000 VL patients will be followed for 4 years, and different treatment arms are included (PM, miltefosine, SSG, and AmBisome®) and monotherapy versus combination therapy.

Dr. J. Alvar noted that every year there are more than 1000 papers reported in peer reviewed journals, but so far only 6 of those were empirical assessments of underreporting. Consequently, WHO made a recent effort to renew estimates of worldwide incidence of leishmaniasis, dividing underreporting into categories of mild, moderate, and severe. Underreporting of VL in India is severe (>4-fold). The estimated worldwide incidence of VL is 202,100 to 389,100 cases per year, in South Asia 162,100 to 313,700, and in East Africa 28,300 to 56,800. However, there are limitations, which is why the paper was published in an open access journal, and can then be used to update the epidemiological information, which is planned for 2013 [1].

Importantly all VL drugs have been incorporated into WHO’s essential drug list, which needs to be followed by updated national guidelines and registration. The price of AmBisome® and Glucantime (an antimonial) was significantly reduced in 2008. Most drugs only have a single producer, which is a problem particularly for miltefosine. Antimonials have access issues: GlaxoSmithKline originally planned to stop production in 2012 and is now negotiating technology transfer, and Albert David’s SSG has quality issues. There are multiple formulations of liposomal or lipid formulations of amphotericin B that need to be evaluated.

A donation of AmBisome® was recently negotiated with Gilead (USA). Countries that have high endemicity and low income and governments that agree with WHO’s VL strategy are eligible to receive the donation. This strategy envisions VL elimination in South Asia by 2020 through the use of AmBisome® 10 mg/kg single dose as first-line treatment for VL in South Asia; and alleviating the consequences of VL in East Africa through compassionate treatment with AmBisome® of moribund, pregnant, and HIV co-infected patients, relapses, and PKDL. Currently, four countries have been identified including Bangladesh, Ethiopia, South Sudan, and Sudan. There are constraints due to the cold-chain requirement and training to administer the intravenous infusion. The planned outcome is to have 100% of VL cases detected and treated in Bangladesh by 2015 (12% of global burden). A total of 52,000 patients will be treated and 10,000 lives saved annually, with a reduction of VL mortality in Africa to <2%. PKDL will be substantially reduced. Implementation will start by the end of 2012. The United Kingdom’s Department for International Development (DFID) will support training, distribution, and active case detection activities.

#### Discussion of control

•Integrated active case detection for VL and PKDL has been ongoing in Bangladesh and India, although in India it is mainly based on information, education, and communication (IEC).

•Case management and guidelines for treatment of PKDL have been discussed and agreed at a WHO meeting in Kolkata in July 2012.

### Reports of working groups

After attendees shared information during 2 days of presentation and discussion sessions, working groups convened for a day to draft recommendations. Overall working group sessions were chaired by Prof. Mohamed Abul Faiz, Prof. Shyam Sundar, and Dr. Jorge Alvar.

### Diagnosis

The working group on diagnosis was chaired by Dr. Monique Wasunna and Prof. Suman Rijal. Validation of diagnosis on a clinical basis is important because parasitological confirmation is not practical in field conditions. This may be a feasible approach in hyperendemic areas (probably 90% of cases will be correctly diagnosed), but in areas with low endemicity where PKDL is not routinely seen, parasitological or molecular confirmation is preferable (also preferred for clinical studies).

**Figure 3:**
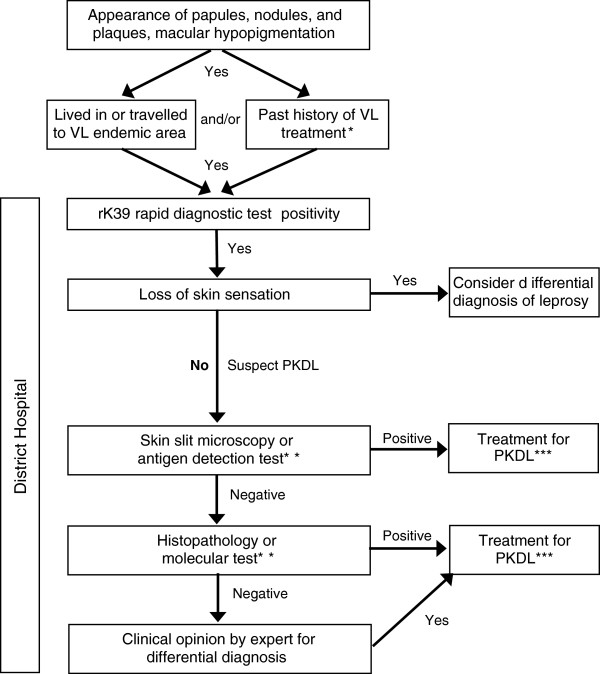
**Desired Algorithm for PKDL Diagnosis (not validated).** Suspect cases are identified based on clinical symptoms, proximity to an endemic region, past VL treatment, and no loss of skin sensation. Confirmation of diagnosis should first be attempted using skin slit smear and parasitology; however, the technique used to obtain the skin slit impacts sensitivity of the method (currently 40-50%). Recommendations for obtaining a skin slit sample will improve sensitivity. *Approximately 10% of PKDL cases present without a history of VL. **Antigen detection and molecular tests are not yet available or are not yet validated. ***The indication for treatment may vary per endemic region.

The group developed a general draft algorithm for diagnosis of PKDL (Figure 3). Suspected cases are defined as those with (1) clinical symptoms, (2) from an endemic region and/or with a past history of VL treatment, and (3) no loss of skin sensation. Exclusion of differential diagnoses (e.g., leprosy) will contribute to more accurate diagnoses of PKDL. Confirmation using slit skin smear and parasitology currently only has 40% - 50% sensitivity, and the working group stated that development of recommendations and training materials on how to obtain a skin slit sample would improve sensitivity of parasitology (microscopy of skin slits is insensitive to the macular form of disease). An antigen detection test such as rK39 to screen for past infection would be ideal. Molecular tests have not yet been standardized for use. The following elements were listed as desired for a test for PKDL primary diagnosis: (1) rapid diagnostic test (<30 minutes to result), (2) antigen detection, (3) >90% sensitive and >95% specific, (4) non-invasive sampling (urine, whole blood, possibly saliva, mucosal swabs), (5) affordable, (6) heat stable (no cold chain), (7) easy to use, and (8) differentiate VL and PKDL. Response to treatment can also be seen as a diagnostic criterion.

The working group defined the following research priorities: (1) define and validate a diagnostic algorithm based on the draft sample shown in Figure 3; (2) evaluate a qPCR test following Standards for Reporting of Diagnostic Accuracy (STARD) guidelines for diagnostic accuracy studies; (3) create a specimen bank for evaluation of new diagnostic tests (type of samples to be discussed); and (4) evaluate other new diagnostics including antigen detection tests (e.g., new Latex agglutination method [KATEX]) and molecular tools (e.g., LAMP). Important additional research priorities include a test of cure and the validation of biomarkers to measure the infectivity of PKDL (replacing xenodiagnosis).

Defined action points included developing training material for sampling methodologies, especially skin slits and arranging a meeting for the diagnostics working group.

### Treatment

The working group on treatment was chaired by Dr. Ahmed Mudawi Musa (not able to attend) and Dr. Koert Ritmeijer. The group determined that regional differences in treatment strategies, public health priorities, parasite response and drug sensitivity, patient numbers, and patient profiles create the need for region-specific Target Product Profiles (TPPs) (Table 1).

**Table 1:** Regional target product profiles

**Target profile**	**South Asia**	**Africa**
Risk:Benefit	Must be very high Parasitological cure must be achieved	No direct associated mortality with symptomatic treatment
Ease of use		Feasible to use at the level of a health clinic
Course of treatment	Very short course of treatment to facilitate compliance	Minimum acceptable treatment for children: one week (similar to antibiotics)
Cold chain requirements	Ideally not cold chain dependent	
Cost factors	Cost is less important than commitment to treatment	
Concomitant infections		With HIV co-infection, no antimonials or monotherapies

In South Asia, PKDL patients are mainly adults, and the priority is public health disease control. The current recommended therapeutic course that meets the TPP is a short course of AmBisome® 5 mg twice per week for 3 weeks. However, the ability to achieve complete cure in macular lesions as well as lack of future infectivity to sandflies is unknown. Tests that were noted above as key for accurate diagnosis are equally important during treatment to assess parasitological cure and lack of infectivity. The infectivity of patients is a cross-cutting issue of importance for treatment as well as diagnosis. Overall focus for treatment in South Asia was on the following: (1) clinical cure of nodular/papular lesions, (2) parasitological cure must be achieved, (3) benefit-risk ratio must be very high, (4) no additional monitoring at primary health care level, (5) treatment compliance is essential to prevent resistance development (very short course treatment), (6) transportation allowance for patients to ensure treatment compliance, (7) ideally non-cold chain dependence, but acceptable, (8) for elimination, cost is less important than commitment, and (9) cost effectiveness analysis.

In Africa, patients are mainly children, and the priority is symptomatic case management. Currently, no drugs fit the desired TPP. Suggested combinations include AmBisome®+miltefosine, miltefosine+PM, or miltefosine+SSG. Overall focus for treatment in Africa was on the following: (1) no direct associated mortality, (2) toxicity should not be higher than VL treatment, (3) no more laboratory monitoring for VL, (4) feasible at health clinic level, (5) minimum acceptable treatment duration for children: 1 week (as for antibiotics), and (6) HIV co-infection: no antimonials and no monotherapy.

As antimonials were phased out of use, miltefosine has been used to treat PKDL in Nepal with better tolerance than is typically seen with VL treatment; to date, 2 patients have relapsed at 12 months. In India, miltefosine treatment resulted in a 93% cure rate at 12 months. In general, the role of miltefosine monotherapy for treatment of PKDL should not be overlooked for individual cases; however, the potential for emergence of resistance, length of time to achieve a cure, and the teratogenic potential of the drug preclude its use for broad public health interventions. The working group recommended that a multicenter, randomized, controlled trial be conducted to evaluate PKDL treatment options. A long-term goal should be to prevent PKDL, and a planned study in Bangladesh that will follow treated VL patients will give insight in which VL treatments result in the lowest rates of PKDL.

Priority research questions include the impact of VL drugs and treatment duration on the pathogenesis of PKDL. A planned study in Bangladesh will assess the relationship between different VL treatments and subsequent PKDL, although the extrapolation of these results to other regions is as yet unknown. The working group emphasized the importance of ensuring complete treatment of VL, which will require a sustained supply of all drugs used to treat VL (including miltefosine, amphotericin B, AmBisome®, or other drugs).

### Pathogenesis

The working group on pathogenesis was chaired by Dr. Poonam Salotra and Prof. Ahmed M El-Hassan (not able to attend). The main unresolved issues from a pathogenesis perspective were identified as (1) why a small percentage of apparently cured VL cases then develop PKDL, (2) whether PKDL is caused by residual parasites or re-infection, (3) why Asia and Africa have differences in incidence, time lapse after VL, and presentation, (4) how the parasite shifts from a visceral to dermal site of infection, and (5) why treatment requires a 3- to 4-fold longer duration of drug therapy.

The working group discussed whether a study on biomarkers could be designed similar to what has been done for VL. The working group discussed work by Dr. Salotra on *in vitro* drug susceptibility before and after use of miltefosine: PKDL isolates became 3–4 times less susceptible to miltefosine after treatment. The group discussed an interest in understanding why areas bitten by sandflies are hands and legs but the PKDL lesions are usually seen on the face; this was within the context of discussion that sandfly bites result in delayed type hypersensitivity, which causes a large accumulation of macrophages around the site. The working group voiced concern that the numbers of patients needed for some suggested studies may not be feasible to obtain (thousands of PKDL patients).

Top priorities identified by this group include (1) genetic analysis of host to identify markers of susceptibility to PKDL, (2) follow up of VL patient immune response to identify immunologic markers predictive of PKDL development, (3) preparation of paired isolates from cases of VL and PKDL, including a repository of parasites and serum, (4) identification of serum markers to monitor progression toward a cure-defined endpoint to treatment, (5) determination of referral laboratories that can confirm diagnosis using histopathology / PCR in microscopy negatives (at least for research studies), (6) whole genome sequence analysis of parasites (African and Asian strains) that will lead to discovery of biomarkers to confirm diagnosis and assess cure, (7) understand why longer duration of treatment (3–4 times) is needed for PKDL versus VL (modify drugs to target skin?), (8) development of an animal model for PKDL, (9) mechanism of hypopigmentation, (10) discover antibodies to sandfly components in PKDL versus VL serum, and (11) nutritional factors (e.g., cholesterol) that may be predictive of host risk of infection.

### Epidemiology and control

The working group on epidemiology and control was chaired by Dr. Dinesh Mondal and Dr. Dia El Naiem. The group determined that all regions need to focus on understanding the exact burden of PKDL using active surveillance and geographic information system (GIS) mapping. Innovative methods such as “little doctor concept” were discussed. Achieving an understanding of the infectiousness of different forms of PKDL as relates to VL infection, asymptomatic cases, oligosymptomatic cases, and HIV co-infected patients (xenodiagnosis to assess infectiousness, validation of biomarkers to replace xenodiagnosis, establishment of when/whether PKDL and VL patients are sterile after treatment) may allow for epidemiologic modeling. This knowledge will also lead to decisions about which patients need treatment and for how long, best methods for preventing transmission (e.g., longlasting treated net [LLTN], focal spraying), and better prediction of outbreaks.

The group discussed timing and methods for active surveillance of PKDL and VL: (1) on a regular basis, including after the elimination goal is reached, (2) case finding around index cases is most effective, (3) health education as an essential component, (4) use of GIS to define microfoci, and (5) if possible, combine with immediate IRS.

The best approach for active case detection (around index cases, camp approach, house-to-house or an incentive-based approach) may depend on the region/context; however, case finding around an index case was generally determined to be most effective by MSF in Fulbaria versus house to house detection. The following measures were considered important for identification of PKDL cases: (1) trained health worker able to recognize lesions, (2) physician to confirm, and (3) epidemiologic link and positive rK39. The group noted that an amount of false positive cases will occur and discussed whether 5% to 10% would be acceptable.

The group suggested the following control strategies: focal spraying in low-endemic areas when a case is detected and blanket spraying in high-endemic areas; regional decisions to actively treat PKDL depending on toxicity of available treatments; and providing free LLTNs to PKDL and VL patients, which may serve as an incentive for self-reporting. A sufficient budget will be essential to assure continuity of spraying.

Overall, the working group determined that the strategy should be similar for South Asia and Africa, except IRS, taking into account the natural history of disease for each area. IRS twice per year significantly reduces the number of cases, but both need to be supervised; the group discussed that VL recurred in sprayed areas.

Two main research priorities, accurate assessment of number of PKDL cases and their infectiousness, were considered equally important toward the goal of control.

### Closing session

The closing session was chaired by Prof. Ed Zijlstra and Dr. Philippe Desjeux. The Rapporteurs report was presented by Dr. M. den Boer and Dr. Sally Ellis. The following research priorities were identified:

#### Epidemiology

•Measure the exact burden of PKDL by active surveillance and GIS mapping

•Measure the infectiousness of different PKDL forms compared to VL/ asymptomatics/ oligosymptomatics/HIV co-infected:

○ Xenodiagnosis to assess infectiousness

○ Validate biomarkers to replace xenodiagnosis

○ Establish when/whether PKDL and VL patients are sterile after treatment

•Epidemiologic modeling (based on the results of infectiousness experiments). If PKDL patients are infectious, this may help to:

○ Decide which PKDL patients need treatment and for how long

○ Determine the best method for prevention: LLTNs, IRS

○ Predict outbreaks

#### Control

•Regular active case detection

○ Training community healthcare workers and strong referral system

○ Health education (IEC and behavior change communication)

•Vector control

○ High endemic areas: blanket spraying

○ Low endemic areas: when a case is detected, focal spraying

•Provide free LLTNs for PKDL and VL patients, which serve at the same time as an incentive for self-reporting of PKDL

•GIS mapping

○ Treatment of PKDL depending on region and toxicity of available treatment

#### Diagnosis

•Validate clinical diagnosis of PKDL

•Develop diagnostic test and test of cure suitable for use in the field: cheap, rapid, antigen-based, heat stable, differentiates between VL and PKDL

•Standardized qPCR method to quantify parasites in different disease and treatment stages

•Develop test of cure

○ Based on antigen detection or qPCR

○ To assess when to stop treatment

○ To monitor for resistance

#### Pathogenesis

•Genetic factors determining development of PKDL (African vs. Asian)

•Immunologic markers to predict outcome of VL treatment and influence co-existing factors such as nutrition and co-infections

•Collect paired isolates from VL and PKDL

•Serum markers for progression

•Referral laboratories are needed

•Genome sequence analysis of African vs. Asian strains

•Explore duration of treatment: why is treatment for PKDL longer than for VL?

•Animal model for PKDL

•Explore the mechanism of hypopigmentation in PKDL

•Explore the role of nutritional status

#### Treatment

•Clinical trials of a short-course regimen

•South Asia: short-course regimen involving AmBisome®

How to assess cure in papular/nodular and macular PKDL?

•Africa: Study new combinations

AmBisome®+miltefosine

Miltefosine+PM

Miltefosine+SSG

•Immunochemotherapy

•Skin penetration of drugs

#### Cross-cutting issues

•Africa/Asia cooperation

•Sandfly colonies for xenodiagnosis

•Sample bank(s): isolates from VL and PKDL cases, repository of parasites, serum, etc.

•Genome sequence analysis African vs. Asian strains

#### Consensus

Priority is to establish infectiousness via biomarkers but since this may take several years, in parallel the following should be prioritized (short term-quick impact):

•First indication of infectiousness via xenodiagnosis

•Validate clinical diagnosis to enable active case surveillance

•Develop a PCR method to confirm PKDL and test of cure so that clinical studies become feasible

•Assessment of real PKDL burden

In conclusion, there is an urgency to continue the momentum created with PKDL integration into the VL elimination program. Awareness of PKDL needs to be raised among scientists, policy makers, health workers, and patients. High-impact study protocols should be developed to investigate the priority research questions. Achieving these goals will require individual research institutes to cooperate and avoid unnecessary repetition or partial answers. A platform for continued scientific exchange is needed to facilitate cooperation between research institutes and the organizations or governments that are implementing control programs.

### Post-meeting update

The colleagues from Sudan, Prof. A. M. El-Hassan, Prof. E. A. G. Khalil, and Dr. A. M. Musa were not able to attend the meeting for reasons beyond their control. These colleagues participated during drafting of the consortium meeting report and provided follow-up information:

•PKDL in Sudan is unique and differs from the disease in Asia in several ways. Thus, what has been discovered in Sudan, particularly with pathogenesis, may not apply to Asian cases. PKDL in Sudan is related etiologically to exposure to UVB light; UVB alters the antigen-presenting capacity of Langerhans cells so that they present leishmania antigen to T reg cells only. This is thought to occur after an initial phase in which CD3 cells arriving in the skin from the spleen after cure of VL are able to activate macrophages and reduce the parasite load. When T reg cells are subsequently activated, there is persistent antigen and few parasites. The T reg cells are not able to activate CD8 cells, which are required to damage macrophages that have antigen in the cytoplasm and cell membrane. This is likely mediated by cell-to-cell contact or elaboration of cytokines, particularly IL-10. Current research is focusing on why and how lesions eventually regress or persist.

•A protocol that investigates sun screens are applied at different stages of PKDL will help determine whether sun screens against UVB will prevent PKDL or ameliorate it and lead to healing.

•Another area of research interest is predictors of healing versus persisting PKDL. Prior research showed that patients with a PKDL history >6 months do not self-heal and require prolonged treatment with SSG. Patients with a PKDL duration <6 months may heal spontaneously or with short courses of drugs. Those who have a positive leishmanin skin reaction or have more IFN-γ than IL-10 in their peripheral blood mononuclear cells do well. Whether IFN- γ may be a better early predictor of favorable outcome than leishmanin requires further study.

•In the past, immunochemotherapy was used to treat PKDL. The first-generation vaccine autoclaved *Leishmania major* and BCG vaccine mixture and ALUM as a stimulant were used along with SSG in persistent PKDL. The duration of treatment was reduced from 2–4 months when SSG alone was used to 40 days with immunochemotherapy. Importing the vaccine has been difficult; local manufacturing of the vaccine in Sudan would be preferable.

•It is unclear whether the parasite causing VL and subsequently PKDL are genetically the same, nor it is known whether they have the same sensitivity to the most frequently used drug SSG.

•LEAP will continue to collaborate with DND*i* to find new drugs or combinations of drugs for VL and PKDL.

•An important objective at the IED is to develop the infrastructure and train young scientists through the project. This will require long-term support.

•This is an in-house part of the agenda for research on leishmaniasis for the next 5 years.

## Consent

We have written permission from those depicted on the images.

## Abbreviations

BCG: Bacille calmette-guérin; BID: Twice per day; BMGF: Bill & Melinda Gates foundation; BPKIHS B.P.: Koirala institute of health sciences Kathmandu, Nepal; CL: Cutaneous leishmaniasis; CpG: Cytosine phosphate Guanine; DAT: Direct agglutination test; DFID: Department for international development; DNDi: Drugs for neglected diseases *initiative*; ELISA: Enzyme-linked immunosorbent assay; GIS: Geographic information system; GLA-SE: Glucopyranosyl lipid adjuvant-stable emulsion; HAART: Highly active anti-retroviral therapy; HIV: Human immunodeficiency virus; ICDDR,B: International centre for diarrhoeal disease research, Bangladesh; ICMR: Indian council of medical research; IDRI: Infectious disease research institute; IEC: Information, education, and communication; IED: Institute of endemic diseases, Khartoum, Sudan; IFN: Interferon; IFN-γR1: IFN-γ receptor gene; IL: Interleukin; IRF: Interferon regulatory factor; IRS: Indoor residual spraying; ITBN: Insecticide-treated bed nets; LAMP: Loop-mediated isothermal amplification; LD: *Leishmania donovani*; LEAP: Leishmaniasis east africa platform; L-FABP: L-fatty-acid-binding protein; LLTN: Longlasting treated net; LST: Leishmanin skin test; MPL-SE: Monophosphoryl lipid a-stable emulsion; MSF: Médecins Sans frontières; NO: Nitric oxide; OWH: Oneworld health; PBMC: Peripheral blood mononuclear cells; PKDL: Post kala-azar dermal leishmaniasis; PM: Paromomycin; qPCR: quantitative polymerase chain reaction; RMRI: Rajendra Memorial Research Institute, Patna, India; RTAG: Regional technical advisory group; SEARO: South-east asia regional office of the WHO; SKKRC: Surya kanta kala-azar Research Center; SSG: Sodium stibogluconate; STARD: Standards for reporting of diagnostic accuracy; Th T: helper cell; TGF: Transforming growth factor; TID: Three times per day; TNF: Tumor necrosis factor; TPP: Target product profile; TSLA: Total soluble *Leishmania* antigen; UT: University of Tokyo; UV: Ultraviolet; VL: Visceral leishmaniasis; WHO: World health organization.

## Competing interests

The authors declare that there are no competing interests.

## Authors’ contributions

All consortium contributors mentioned in the manuscript reviewed and approved the final version of the Consortium Report upon which the manuscript was based. All authors reviewed and approved the final version of the manuscript.

## Supplementary Material

Additional file 1 The meeting program. Click here for file

Additional file 2 List of participants. Click here for file
