# Supplementary material for: Report of the Post Kala-Azar Dermal Leishmaniasis (PKDL) consortium meeting, New Delhi, India, 27–29 June 2012
Source: Parasit Vectors. 2013 Jul 2;6:196. doi: 10.1186/1756-3305-6-196 (PMC3733610; doi:10.1186/1756-3305-6-196)
Supplement: Additional file 1 — The meeting program. [file 1756-3305-6-196-S1.docx]

**Post Kala-Azar Dermal Leishmaniasis (PKDL) Consortium Meeting**

**New Delhi, June 27-29, 2012**

**PROGRAM**

WEDNESDAY, JUNE 27

**OPENING SESSION**

**Chairs: Dr. Philippe Desjeux, Prof. Ed Zijlstra**

8:30 – 9:00 Welcome

1) Indian Council of Medical Research (Dr VM. Katoch, Director, ICMR), 2) Ministry of Health of India, National Vector Borne Diseases Control Program (Dr AC. Dhariwal, Director NVBDCP), 3) OneWorld Health / PATH (Dr Tarun Vij, Country Program Leader, PATH), 4) Drugs for Neglected Diseases *initiative* (Dr Nathalie Strub-Wohrgraft, DND*i*), 5) the World Health Organization (Dr J. Alvar, NTDs, WHO, Geneva), 6) the Bill & Melinda Gates Foundation (Dr Lalit Kant, BMGF)

9:00 – 9:30 PKDL consortium

Dr. Philippe Desjeux, Prof. Ed Zijlstra

**SESSION 1: EPIDEMIOLOGY**

**Chairs: Prof. Ahmed M. El-Hassan, Prof. NK Ganguly**

9:30 – 9:50 India

Prof. CP Thakur

9:50 – 10:10 Bangladesh

Prof. Mohamed Abul Faiz

10:10 – 10:30 Nepal

Prof. Suman Rijal

10:30 – 11:00 Discussion

***Coffee break 11:00 – 11:30***

**Chairs: Prof. Ridwanur Rahman, Dr. Monique Wasunna**

11:30 – 11:50 Sudan

Prof. Ed Zijlstra

11:50 – 12:10 Ethiopia

Prof. Asrat Hailu

12:10 – 12:30 Entomology review: global

Dr. Dia El Naiem

12:30 – 13:00 Discussion

***Lunch 13:00 – 14:00***

**SESSION 2: DIAGNOSIS**

**Chairs: Prof. Asrat Hailu, Dr. Emily Adams**

14:00 – 14:20 Sudan

Dr. Ahmed Mudawi Musa

14:20 – 14:40 Nepal

Prof. Suman Rijal

14:40 – 15:00 Bangladesh

Prof. Yoshitsugu Matsumoto, Dr. Eisei Noiri

15:00 – 15:10 India

Dr. Poonam Salotra

15:10 – 15:30 Discussion

***Tea break 15:30 – 16:00***

**SESSION 3: PATHOGENESIS**

**Chairs: Prof. Farrokh Modabber, Dr. Pradeep Das**

16:00 – 16:20 Pathology, parasites, genetics

Prof. Ahmed M. El-Hassan

16:20 – 16:40 Immunology – India

Dr. Poonam Salotra

16:40 – 17:00 Immunology – Sudan

Dr. Ahmed Mudawi Musa

17:00 – 17:20 Role of drugs

Dr. Epco Hasker

17:20 – 17:50 Discussion

THURSDAY, JUNE 28

**SESSION 4: CLINICAL PRESENTATION**

**Chairs: Dr. Jorge Alvar, Dr. Dinesh Mondal**

8:30 – 8:50 Sudan

Dr. Ahmed Mudawi Musa

8:50 – 9:10 India

Prof. V Ramesh

9:10 – 9:30 Bangladesh

Prof. Ridwanur Rahman

9:30 – 9:50 PKDL in immunocompromised patients

Prof. Ed Zijlstra

9:50 – 10:20 Discussion

***Coffee break 10:20 – 10:40***

**SESSION 5: TREATMENT**

**Chairs: Dr. VM Katoch**

10:40 – 11:00 Africa

Dr. Ahmed Mudawi Musa

11:00 – 11:20 India

Prof. V Ramesh

11:20 – 11:40 Bangladesh

Dr. Dinesh Mondal

11:40 – 12:00 Médecins Sans Frontières (MSF): field experience

Dr. Koert Ritmeijer and MSF Spain

12:00 – 12:30 Immune modulation: outlook

Prof. Farrokh Modabber

12:30 – 13:00 Discussion

***Lunch 13:00 – 14:00***

**SESSION 6: CONTROL**

**Chairs: Dr. AC Dhariwal, Prof. Be-Nazir Ahmed, Dr. Garib Das Thakur**

14:00 – 14:30 VL, PKDL

Dr. Philippe Desjeux, Dr. Jorge Alvar

14:30 – 15:00 Discussion

**WORKING GROUPS**

15:00 – 18:00 Diagnosis

Chairs: Dr Monique Wasunna, Prof. Suman Rijal

Treatment

Chairs: Dr. Ahmed Mudawi Musa, Dr. Koert Ritmeijer

Pathogenesis

Chairs: Dr. Poonam Salotra, Prof. Ahmed M. El-Hassan

Epidemiology/ control

Chairs: Dr. Dinesh Mondal, Dr. Dia El Naiem

***Conference dinner 19:00 – 22:00***

FRIDAY, JUNE 29

**REPORTS OF WORKING GROUPS**

**Chairs: Prof. Mohamed Abul Faiz, Prof. Shyam Sundar**

8:30 – 9:00 Diagnosis

9:00 – 9:30 Discussion

9:30 – 10:00 Treatment

10:00 – 10:30 Discussion

***Coffee break 10:30 – 11:00***

**Chairs: Dr. Margriet den Boer**

11:00 – 11:30 Pathogenesis

11:30 – 12:00 Discussion

12:00 – 12:30 Epidemiology/ control

12:30 – 13:00 Discussion

***Lunch 13:00 – 14:00***

**CLOSING SESSION**

**Chairs: Prof. Ed Zijlstra, Dr. Philippe Desjeux, Prof. Ahmed M. El-Hassan**

14:00 – 16:00 Rapporteurs commentary (Dr. Margriet den Boer and Dr. Sally Ellis), way forward, open discussion
