# Supplementary material for: Report of the Post Kala-Azar Dermal Leishmaniasis (PKDL) consortium meeting, New Delhi, India, 27–29 June 2012
Source: Parasit Vectors. 2013 Jul 2;6:196. doi: 10.1186/1756-3305-6-196 (PMC3733610; doi:10.1186/1756-3305-6-196)
Supplement: Additional file 2 — List of participants. [file 1756-3305-6-196-S2.docx]

**Post Kala-Azar Dermal Leishmaniasis (PKDL) Consortium Meeting**

**New Delhi, June 27-29, 2012**

**PARTICIPANTS**

**BANGLADESH**

**Prof. Be-Nazir Ahmed, Director, Disease Control (DC) and Line Director, CDC, Directorate General of Health Services (DGHS), Dhaka**

**Dr. Mannan Bangali, World Health Organization (WHO), Dhaka**

**Dr. Ahmed Mohamed Elshafie, Médecins Sans Frontières (MSF)-OCA, Dhaka**

**Prof. Mohamed Abul Faiz, Dhaka**

**Dr. Ulrika Markin, MSF-OCA, Fulbaria sub-district, Mymensingh district**

**Dr. Dinesh Mondal, International Centre for Diarrhoeal Disease Research, Bangladesh (ICDDR,B), Dhaka**

**Dr. Shah Golam Nabi, Kala-Azar Elimination Program, DGHS, Dhaka**

**Prof. Md. Ridwanur Rahman, Begum Khaleda Zia Medical College, Dhaka**

**BELGIUM**

**Prof. Marleen Boelaert, Institute of Tropical Medicine (ITM), Antwerpen, Belgium**

**Dr. Epco Hasker,Institute of Tropical Medicine (ITM), Antwerpen, Belgium**

**BILL & MELINDA GATES FOUNDATION (INDIA)**

**Dr. Lalit Kant, BMGF, New Delhi**

**Dr. Anand Sinha, BMGF, New Delhi**

**DRUGS FOR NEGLECTED DISEASES INITIATIVE (DND*i*)**

**Ms. Sally Ellis, Geneva, Switzerland**

**Prof. Farrokh Modabber, Geneva, Switzerland**

**Dr. Bhawna Sharma, New Delhi, India**

**Dr. Nathalie Strub-Wohrgraft, Geneva, Switzerland**

**Prof. Ed Zijlstra, Geneva, Switzerland**

**ETHIOPIA**

**Prof. Asrat Hailu, Addis Ababa University, Addis Ababa**

**FOUNDATION FOR INNOVATIVE NEW DIAGNOSTICS (FIND)**

**Dr. Audrey Albertini, Geneva, Switzerland**

**Dr. CN Paramasivan, New Delhi, India**

**INDIA**

**Dr Sujit Bhattacharya, Society for Applied Studies, Kolkata**

**Dr. Sakip Bursa, MSF-OCA, Spain, New Delhi**

**Dr. Pradeep Das, Director, Rajendra Memorial Research Institute (RMRI), Indian Council on Medical Research (ICMR), Patna**

**Dr. Ajay Chandra Dhariwal, Director, National Vector Borne Disease Control Program (NVBDCP), New Delhi**

**Prof. NK. Ganguly, National Institute of Immunology, New Delhi**

**Dr. VM. Katoch*, Director General, ICMR, New Delhi***

**Prof. V. Ramesh, Safdarjang Hospital, New Delhi**

**Dr. Poonam Salotra, Institute of Pathology, ICMR, New Delhi**

**Prof. Shyam Sundar, Banaras Hindu University (BHU), Varanasi**

**Prof. CP. Thakur, India Patna Medical College, Patna**

**JAPAN**

**Prof. Yoshitsugu Matsumoto, University of Tokyo, Tokyo**

**Dr. Eisei Noiri, University of Tokyo, Tokyo**

**KENYA**

**Dr. Monique Wasunna, the Kenya Medical Research Institute (KEMRI); DND*i* (Africa), Nairobi**

**MEDECINS SANS FRONTIERES - HOLLAND**

**Dr. Koert Ritmeijer, Amsterdam, The Netherlands**

**NEPAL**

**Prof. Suman Rijal, BP Koirala Institute of Health Sciences (BPKIHS), Dharan, Morang**

**Dr. Nihal Singh, WHO, Kathmandu**

**Dr. Garib Das Thakur, Director, Epidemiology and Disease Control Division (EDCD), Ministry of Health and Population, Kathmandu**

**ONEWORLD HEALTH (OWH), AFFILIATE OF PATH**

**Dr. Philippe Desjeux, Divonne, France**

**Dr. Pritu Dhalaria, Delhi, India**

**Dr. Raj Shankar Ghosh, Delhi, India**

**Dr. Syed Hassan, Patna, India**

**Dr. Sonali Kochhar, Delhi, India**

**Dr. Amresh Kumar, Delhi, India**

**SUDAN**

**Prof. Ahmed M. El-Hassan, Institute of Endemic Diseases (IED), Khartoum**

**Dr. Ahmed Mudawi Musa, Institute of Endemic Diseases (IED), Khartoum**

**THE NETHERLANDS**

**Dr. Emily Adams, Royal Tropical Institute (RTI), Amsterdam**

**Dr. Thomas Dorlo, Academic Medical Centre, Amsterdam**

**USA**

**Dr. Caryn Bern, University of California, San Francisco (UCSF)**

**Dr. Dia El Naiem, University of Maryland, Princess Ann, Salisbury**

**WHO/HEADQUARTERS**

**Dr. Jorge Alvar, Department of Control of Neglected Tropical Diseases (NTDs), Geneva, Switzerland**

**Dr. Byron Arana, TDR, Geneva, Switzerland**

**Dr. Daniel Argaw Dagne, Department of Control of Neglected tropical Diseases (NTDs), Geneva, Switzerland**

**WHO/SOUTH-EAST ASIA REGIONAL OFFICE (SEARO)**

**Dr. C.R. Revankar, Vector-borne & NTDs Control, New Delhi, India**
